# Reply to: Critical COVID-19 and neurological dysfunction - a direct comparative analysis between SARS-CoV-2 and other infectious pathogens

**DOI:** 10.5935/2965-2774.20230383resp-en

**Published:** 2023

**Authors:** Ana Teixeira-Vaz, José Artur Paiva

**Affiliations:** 1 Physical Medicine and Rehabilitation Department, Centro Hospitalar Universitário de São João, Faculdade de Medicina, Universidade do Porto - Porto, Portugal; 2 Intensive Care Medicine Department, Centro Hospitalar Universitário de São João, Faculdade de Medicina, Universidade do Porto - Porto, Portugal

## TO THE EDITOR

The authors thank Dr. Magoon and Dr. Suresh for the letter and for their interest in our study “Critical COVID-19 and neurological dysfunction - a direct comparative analysis between SARS-CoV-2 and other infectious pathogens”.^(1)^

We start by highlighting the importance and pertinence of the first comment in the letter. Indeed, in our analysis, COVID-19 patients spent significantly more days under sedoanalgesia, despite the absence of a significant association between this variable and the presence of neurological dysfunction. Therefore, it is unlikely that this variable could have biased our results. However, we have not performed an in-depth characterization of the nature of the sedation of these patients; nonetheless, we must point out that, in our center, there is a protocol for critical care patients sedation using the ABCDEF bundle as a standard of care.^(2)^

Furthermore, the letter highlighted the importance of evaluating the possible role of preexisting alcoholic status as well as the effect of using antipsychotics on the relationship between sedation and the presence of delirium. We acknowledge the importance of this topic. In our analysis, we retrospectively collected data regarding all included patients’ comorbidities, including psychiatric disorders (which included history of substance abuse). Nonetheless, we did not find a significant difference in the rates of psychiatric disorders between COVID-19 and non-COVID-19 critically ill patients, and we did not find a significant effect of those comorbidities on neurological complication rates.

Finally, we fully agree that the findings by Saxena et al.^(3)^ are relevant and that laboratory biomarkers are important for neuro-prognostication. Nonetheless, the analysis of inflammatory biomarkers, namely, fibrinogen levels, is beyond the scope of our study. Moreover, the abovementioned study was published more than 2 years after our study was designed and after recruitment started. Nevertheless, it would be useful to perform a detailed analysis of the impact of different grades of inflammation on SARS-CoV-2 neuro-prognostication as well as an analysis of the utility and cost-effectiveness of laboratory biomarkers for predicting neurological outcomes in this population. Hopefully, future clinical investigations will examine these issues.
